# Enterotomy on a Bitch

**Published:** 1895-11

**Authors:** Cecil French

**Affiliations:** The Washington (D. C.) Canine Infirmary


### REPORTS OF CASES.

### ENTEROTOMY ON A BITCH.

BY CECIL FRENCH, D.V.S.,
THE WASHINGTON (D. C.) CANINE INFIRMARY.

On Tuesday, September 17th, a valuable dachshund bitch, four months old, was brought to the infirmary manifesting the following symptoms: listlessness, frequent vomiting with expulsion of several ascarides (marginata) per orem, temperature slightly subnormal, pulse quick and wanting in tone, and pronounced distention of the epigastric region. I was further informed by the owner that the little creature had been in that condition since the preceding Friday. On inquiring as to the nature of her habitation and diet, I was told she had the run of a country estate, would eat almost anything, and had been an incorrigible little marauder on the young turkeys.

The abdomen was carefully palpated without feeling anything unusual. A diagnosis of duodenal obstruction from an impacted mass of worms or turkey-bone was made, subject to further developments.

Several small doses of hydrocyanic acid were given with the object of inducing the stomach to retain laxative medicines. The next morning the following vermicide in soft elastic capsular form was administered: santonin, jalap, resin, and calomel, together with twenty minims of castor oil.

In half an hour part of this was vomited with a few ascarides, and later *one* worm of this species defecated. Previously to this copious enemata had been given and the bowel unloaded, but the epigastrium still remained distended. A little nourishment was poured down the throat, but immediately returned. It was then decided to let the proximal end of the alimentary tract rest and await further developments. In the meantime enemata of milk predigested with extract of pancreas were frequently given, as the tissues were rapidly wasting. This seemed to brighten the patient considerably.

Friday morning the epigastric distention had disappeared and a hard obstruction like a peach-stone (which it later proved to be) could be distinctly made out in the hypogastric part of the abdominal cavity. A little meat was then readily eaten and a further dose of castor oil with cascara sagrada was kept on the stomach. I now hoped that the obstruction, having moved so far, would ultimately pass. Saturday at noon, on pressing on the obstruction, great pain was manifested and the general condition appeared worse. Having consulted with my friends, Drs. Richardson, of the sister profession, and Adamson, my esteemed *confrère*, an operation was deemed essential to save the life of the patient. As an anæsthetic, a hypodermic injection of morphine sulphate gr. ¼ with atropine sulphate gr. $\frac{1}{150}$ was given and the flank operation performed, and the obstructing peach-stone quickly removed from the ileum. The intestinal wall at the seat of obstruction was completely paralyzed and highly inflamed. Ordinary gut ligatures were used and the two mucous surfaces brought into tight apposition. The condition of the dilated inflamed portion was such that the Lembert suture through the serous coats would have been inappropriate. Complete recovery quickly followed.

No doubt the stone first became lodged in the duodenum or upper portion of the jejunum and produced the vomiting. Its presence or the extraordinary contractions consequent on the same drove the worms into the stomach, from whence they were speedily ejected.

